# Genetic diversity in Cyatheaceae in a stressful changing climate: a multi-omics review of adaptive evolution and conservation

**DOI:** 10.3389/fgene.2026.1791407

**Published:** 2026-03-18

**Authors:** Tengmin Li, Hanqing Cong, Dandan Rao, Erhuan Wu, Yutong Cui, Rongcun Gan, M. Nasir Khan, Yu Chen

**Affiliations:** 1 Hainan Academy of Forestry (Hainan Academy of Mangrove), Haikou, China; 2 Key Laboratory of Tropical Forestry Resources Monitoring and Application of Hainan Province, Haikou, China; 3 Tropical Crops Genetic Resources Institute, Chinese Academy of Tropical Agriculture Sciences, Haikou, China; 4 Renewable Energy and Environmental Technology Center, University of Tabuk, Tabuk, Saudi Arabia

**Keywords:** conservation genetics, Cyatheaceae, genetic diversity, genomics, transcriptomics

## Abstract

*Cyatheaceae*, assigned to the fern order *Eufilicales*, is one of the most ancient extant lineages of arborescent tree-ferns and is widely distributed across tropical, subtropical, and parts of the temperate Southern Hemisphere. This group is ecologically, scientifically, and medicinally valuable. It serves as a critical bioindicator of forest ecosystem stability, provides an ideal model system for investigating adaptive evolution and stress response mechanisms in plants, and produces secondary metabolites with promising pharmaceutical applications. Recent advances in modern molecular biology have markedly promoted omics-based research on the family. Here, we systematically review multi-omics achievements—genomics, transcriptomics, and related fields—with emphasis on how sequencing technologies and functional genes have elucidated genetic diversity, cryptic lineage divergence, and adaptive evolutionary mechanisms in response to biotic and abiotic stressors, including climate change, habitat fragmentation, and interspecific competition. We summarize current conservation applications, highlight core problems such as taxonomic and geographical sampling bias and insufficient multi-omics integration, and propose future research directions so as to provide a scientific basis for the systematic conservation, sustainable utilization, and in-depth study of the genetic resources of Cyatheaceae under increasing environmental pressures.

## 1 Introduction

Pteridophyta contains approximately 12,000 species and is an important component of terrestrial ecosystems (Wu et al., 1993). The order *Eufilicales* comprises 47 families, including *Acrostichaceae*, Dicksoniaceae, Cyatheaceae, and *Platyceriaceae*, among which the family Cyatheaceae represents a core lineage. It holds particular phylogenetic and ecological research significance due to its unique arborescent habit—possessing a tall, erect, woody stem, which is the only extant fern with such a tree-like form—as well as its relatively complete fossil record and evolutionary continuity. As the core family of Cyatheales, Cyatheaceae is the only extant group of tree-like ferns with tall, erect, lignified trunks (Wang, 1982); fossil records trace back to the Late Jurassic, c. 180 million years ago (Fu, 1991; Sosa et al., 2016), when it formed extensive forest communities together with cycads (*Cycas revoluta*), araucarias (*Araucaria cunninghamii*) and ginkgo (*Ginkgo biloba*) (Zhou et al., 2008). Its long evolutionary history provides key clues for deciphering palaeofloristic succession, adaptive evolution of land plants, and mechanisms of climate-change response (Dong, 2024; Korall et al., 2007). Globally, the family comprises nine genera and c. 690 species, mainly in tropical, subtropical, and parts of the temperate Southern Hemisphere; Central America (c. 340 spp.) and the Asia–Pacific region (c. 280 spp.) are the principal centers of diversity, whereas Africa is relatively depauperated (c. 70 spp.) (Dong, 2024; Loiseau et al., 2020). China is an important modern distribution center, with 3 genera, 14 species, and 2 varieties recorded. Its populations are concentrated in warm, humid forest ecosystems in the south of the country (Dong, 2024; He et al., 2022), among which *Gymnosphaera saxicola* and *G. austroyunnanensis* are rare Chinese endemics (Dong, 2024; Lv et al., 2018). Wild populations have declined sharply because of Quaternary climatic oscillations and human disturbance, and their conservation status has been adjusted repeatedly (Wang, 1993; Yu, 1999; Mo and Liu, 2004; National Forestry and Grassland Administration & Ministry of Agriculture and Rural Affairs, 2021). The value of Cyatheaceae is reflected in several dimensions. Ecologically, population dynamics serve as a biological indicator for assessing the stability of tropical and subtropical forest ecosystems (Xie et al., 2022; Ranil et al., 2017). Scientifically, the unique arborescent habit and vascular-system evolutionary pattern make it an ideal model for exploring plant morphological evolution and environmental-adaptation mechanisms (Huang, 2022; Huang et al., 2022). In applied terms, secondary metabolites exhibit significant antioxidant, anti-inflammatory, and anti-cancer bioactivities, indicating potential medicinal value (Narayanan and Marimuthu, 2016; Janakiraman et al., 2023; Goh et al., 2007).

**FIGURE 1:**
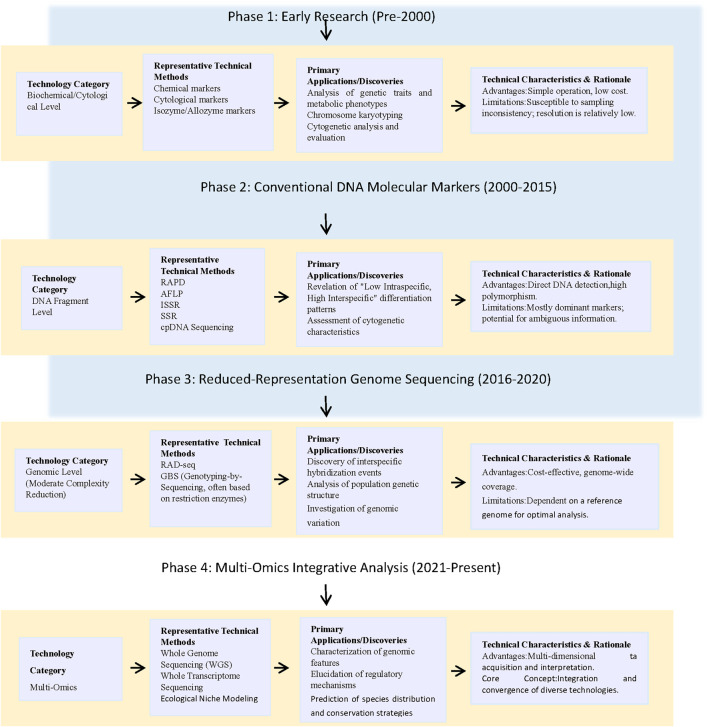
Workflow of the main technical approaches for genetic diversity studies in Cyatheaceae plants.

Genetic diversity is the basis for species to cope with environmental change and to maintain long-term evolutionary adaptive potential. It is also the core criterion for endangered-species conservation strategies (Zhan, 2022a; Pelosi and Sessa, 2021; Frankham et al., 2002). Research was initially constrained by technology, depending primarily on morphological and cytological observations that hindered the accurate quantification of genetic variation. This limitation was overcome by the advent of molecular biology techniques, including SSR and ISSR markers and cpDNA fragment sequencing, which propelled the field to the molecular level (Schwartsburd et al., 2015; Wang T. et al., 2021; Ma et al., 2020; Dong et al., 2019). In recent years, the cross-integration of genomics, transcriptomics, and other multi-omics with ecological-niche modelling has achieved a transition from “single-gene/fragment” to “whole-genome level”, from “genetic-structure description” to “functional-adaptation analysis”, and from “pattern speculation” to “empirical correlation verification”. At present, domestic and foreign scholars have carried out systematic studies on representative genera and species such as *Alsophila spinulosa* and *Sphaeropteris brunoniana*, achieving breakthroughs in chloroplast-genome evolution and cryptic-diversity discovery. However, problems such as highly unbalanced taxonomic and geographical sampling, insufficient in-depth multi-omics collaborative analysis, and lagging translation of research results into conservation practice still exist (Yi et al., 2023; Wang et al., 2019; Krishnan and Rekha, 2021). This study aims to systematically review the advancements in multi-omics research on Cyatheaceae. Unlike previous syntheses that either focused on single-omics snapshots (e.g., Su et al., 2005; Huang, 2022) or summarized regional conservation statuses (Wang and Guan, 2011a), the present review is the first to couple genomics, transcriptomics, proteomics, metabolomics and microbiomics under one framework and to explicitly link each layer to actionable conservation units for the entire family (see Section 6; Figure 1). Then conduct an integrated assessment of the current status of its genetic-diversity and conservation strategies, with a particular focus on deciphering the genetic and molecular basis of its adaptation to biotic (e.g., pathogens, competition) and abiotic (e.g., climate change, habitat alteration) stressors. This will provide a scientific basis for the systematic conservation and in-depth investigation of this ancient lineage in an increasingly stressful global environment.

To avoid confusion, taxonomic abbreviations used hereafter (Alsophila s.l., Alsophila s.s and Gymnosphaera) are clarified in Table 1.

**TABLE 1:** Taxonomic abbreviations used in this review.

Abbreviation	Meaning	Taxonomic scope
*Alsophila s.l.*	*Alsophila sensu lato* (broad sense)	Includes *Gymnosphaera*
*Alsophila s.s.*	*Alsophila sensu stricto* (strict sense)	Excludes *Gymnosphaera*
*Gymnosphaera*	Genus resurrected from within *Alsophila s.l.*	Monophyletic clade with marginal scales (Dong and Zuo, 2018)

## 2 Research techniques and methodological evolution in genetic-diversity studies of Cyatheaceae

### 2.1 Early research stage (before 2000)

Morphological markers inferred genetic differences by measuring variations in leaf shape, spore size, and other morphological characters. Xie et al. (2022) found that the mortality rate of first-age-class individuals of *Alsophila spinulosa* in Diaoluo Mountain, Hainan, was high, inferring that young individuals had poor genetic adaptability. Krishnan and Rekha (2021) identified three geographical ecotypes of Cyatheaceae in the Western Ghats of India through leaf-morphological variation; Morigengaowa et al. (2019) demonstrated that the morphological differences between Hainan’s *Sphaeropteris hainanensis* and *Sphaeropteris brunoniana* align closely with their genetic clustering, supporting species delimitation according to the “integrated species concept”. Song (2004) found that morphological indexes such as plant height and ground diameter of *A. spinulosa* differed significantly among populations in different habitats and were correlated with genetic-diversity levels. This method is convenient to operate but is susceptible to environmental interference, making it difficult to distinguish phenotypic from genetic variation limited resolution.

Cytological markers took chromosome number and karyotype characters as the core. Chromosome numbers in Cyatheaceae are highly conserved, generally diploid (2*n* = 138), with gametophyte chromosome number *n* = 69 (Manton, 1950). Wang T. et al. (2021) and the genome sequencing of Huang et al. (2022) both confirmed this conclusion in studies on *Gymnosphaera podophylla* and other species. Karyotype analysis shows that sub-telocentric (st) and telocentric (t) chromosomes predominate. Wang et al. (1997) found that the karyotype formula of *A. spinulosa* in Guizhou was 2*n* = 2*X* = 16 m + 2 sm + 86 st + 34 t, with karyotype type 1B, indicating that chromosome structure is stable but highly asymmetric. Wang et al. (2020) confirmed that *G. metteniana* is an allotetraploid (2*n* = 276) that originated from hybridization between the diploid parents *G. denticulata* and *G. gigantea*, corroborating the role of polyploidization in evolution.

Isozyme/allozyme markers were the first biochemical molecular markers to be widely used. Pelosi and Sessa (2021) pointed out that about 62% of genetic studies on Cyatheaceae before 2000 adopted this technique. Early studies showed that the percentage of polymorphic loci in *Alsophila* ranged from 22.03% to 27.97%, and gene diversity (Ht) ranged from 0.059 to 0.077 (Wang et al., 2004). The percentage of polymorphic loci in Taiwanese *A. spinulosa* reached 34.5%, and expected heterozygosity (He) was 0.141 (Cheng et al., 2008), inferring that this was related to population size and outcrossing breeding strategy. This marker is low-cost, but the number of polymorphic loci is limited, resolution is low, and enzyme activity is susceptible to environmental and developmental-stage interference. Conant and DeMaggio (1989) applied Western blotting to analyze fern spore-storage proteins, providing biochemical evidence for inferring phylogenetic relationships among distant lineages.

### 2.2 Traditional DNA-molecular-marker stage (2000–2015)

Randomly Amplified Polymorphic (RAPD) markers were widely used because of their simple operation, and the fact that no prior genome sequence was required (Wang et al., 2004; Ewel, 1998; Wang, 2002; Huang and Wang, 2003; Li et al., 2002) revealed the character of “low within-population diversity—high among-population differentiation” in Hainan and Guangdong populations of *A. spinulosa*; Zeng et al. divided six Cyatheaceae species into three genetic groups by RAPD (Huang and Wang, 2003). However, this marker is dominant, cannot distinguish homozygotes from heterozygotes, has poor experimental repeatability, and is sensitive to reaction conditions such as Mg^2+^ concentration, primer concentration, and annealing temperature in the PCR system (Wang et al., 2004; Cheng et al., 2008; Wang, 2002).

Amplified fragment length polymorphism (AFLP) markers combine high polymorphism with wide genome coverage. Wang and Guan (2011b) analyzed 10 *S. brunoniana* populations from China and Laos and showed that their genetic diversity was high (Ht = 0.333, Hsp = 0.499), but differentiation among populations was low (Gst = 0.16, φST = 0.12, and (Hsp–Hpop)/Hsp = 0.16). The limitation of this marker is that it is dominantly inherited and cannot effectively distinguish homozygotes from heterozygotes.

Inter-Simple Sequence Repeat (ISSR) markers combine operational simplicity with low cost. Wang et al. (2012) obtained 74 polymorphic bands in an analysis of 7 *A. spinulosa* populations in China, confirming that it has medium-level genetic diversity and that breeding strategy regulates genetic diversity. Li et al. (2010) reported in his study of *A. costularis* populations in Dawei Mountain, Pingbian, Yunnan, that the percentage of polymorphic loci was 62.87%–73.48% and Nei’s gene diversity reached 0.2614–0.2832, with weak differentiation among populations (Gst = 0.0701). This marker is likewise dominantly inherited and has low primer universality.

Simple Sequence Repeat (SSR) markers are co-dominantly inherited, highly polymorphic, and highly repeatable, and are the “gold-standard” technique for dissecting fine-scale population genetic structure. Yang and Wang (2025) developed 40 SSR primers for *S. brunoniana*, of which 23 pairs showed high polymorphism (polymorphic-band ratio 96.13%) and could effectively delimit genetic populations. Nazareno et al. (2013) developed 11 microsatellite primer pairs for the Brazilian endemic endangered species *Dicksonia sellowiana* for evaluating the impact of rainforest fragmentation; Ramírez-Barahona and Eguiarte (2015) found that *Alsophila firma* populations exhibit strong spatial genetic structure (SGS) and that the intensity of SGS in ferns is generally higher than in seed plants. The limitation of this marker is that species-specific primers must be developed in advance, entailing high initial cost and time consumption.

Chloroplast DNA (cpDNA) sequence markers (atpB-rbcL, trnL-trnF, *etc.*) are maternally inherited and structurally conserved, making them ideal tools for studying plant phylogeny and phylogeography. Su et al. (2005) found, based on cpDNA trnL-F sequences, that Hainan and Guangdong populations of *G. podophylla* share core haplotypes and that spore-dispersal pathways reflect only maternal genetic contribution; Chen X. et al. (2024) confirmed that the cpDNA genome structure of *A. spinulosa* is conserved (GC content 40.44%) and that the atpB-rbcL non-coding region is suitable for analyzing genetic differentiation among populations. The genetic differentiation between the Hainan and mainland populations of *A. spinulosa* was pronounced, with fixation index (*F*_*ST*_) values ranging from 0.92 to 0.95, indicating that the Qiongzhou Strait acting as a key geographical barrier (Su et al., 2005; Su et al., 2004a), whereas no significant differentiation was detected among *G. podophylla* populations spanning the strait, inferred to be related to spore dispersal promoted by glacial land bridges (Su et al., 2005). This marker can reveal population historical dynamics, but it reflects only maternal genetic contribution, and the information content of a single fragment is limited.

Fourier-transform infrared spectroscopy (FT-IR) forms a biochemical “fingerprint” through characteristic absorption peaks; Janakiraman and Johnson (2015) confirmed that it can serve as an indirect indicator of genetic diversity, the technique being rapid, efficient, and non-destructive, but the results are susceptible to environmental influence, and its application in quantitative analysis is limited.

### 2.3 Genome-reduced-sequencing stage (2016–2020)

Second-generation sequencing (NGS) technologies have promoted the application of genome-reduced-sequencing methods such as RAD-seq and GBS, which can acquire thousands to tens of thousands of SNP loci in a single run, improving research resolution and genome-wide coverage (Zhou et al., 2009). Yu (1999) used RAD-seq to analyse 16 Cyatheaceae species from China and Vietnam and found that distant hybridization among species and genera was common. Morigengaowa et al. (2019) used GBS technology based on 18,319 nuclear SNP loci to reveal the genetic-structure differentiation of *Sphaeropteris* populations in Hainan, Yunnan, and Guangxi, the Guangxi population being a hybrid product of Hainan and Yunnan populations. Huang et al. (2022) deciphered the *A. spinulosa* genome (6.2 Gb), increasing the RAD-seq alignment rate from 45% to 78%. This technology pushes research to the whole-genome level, but it is dependent on the quality of the reference genome, and the data-analysis workflow is complex.

### 2.4 Multi-omics and integration-analysis stage (2021–present)

Breakthroughs in whole-genome sequencing technology have provided fundamental support for dissecting core genetic mechanisms. Huang et al. (2022) completed the first chromosome-level genome assembly of *A. spinulosa* (6.23 Gb), identifying a unique phenolic compound—alsophilin—and revealing that the lineage experienced two genetic bottleneck events. Wei et al. (2025) discovered, through comparative genomics, that Cyatheaceae underwent a whole-genome duplication (WGD) event c. 154 million years ago, retaining stress-response genes and enabling functional divergence of duplicated genes; Huang et al. (2025) identified 186 *bHLH* genes at the whole-genome level in *A. spinulosa*, which play an important role in lignin biosynthesis and provide clues to the mechanism of arborescent stem formation.

Whole-transcriptome sequencing has become the core means for functional-gene mining. Hong et al. (2022) carried out whole-transcriptome sequencing of roots, stipes, and fronds of *A. spinulosa*, constructed a non-redundant reference transcriptome, found enrichment of PPR genes and retrotransposon-encoded genes, and high expression of stress-inducible ASR genes. Shi et al. (2024) obtained high-quality unigene sequences of *A. spinulosa*, annotated 26,213 homologous genes in the NR database, and developed 8,996 SSR markers. Peng et al. (2024) revealed the environmental-adaptation mechanisms of *S. brunoniana* and *Alsophila latebrosa* through multi-organ transcriptome and chloroplast-gene analyses.

Integration of ecological-niche modelling with genetic diversity has built a bridge between macro-scale distribution and micro-scale genetics. Wei et al. (2021) integrated niche modelling with spore-development experiments and found that extreme low temperature (mean annual temperature ≥18 °C), temperature stability (seasonal variation ≤3.5 °C), and south-facing slopes were the key factors influencing the distribution of *Sphaeropteris lepifera*, proposing that core areas such as Nan’ao Island, Shantou, Guangdong should be prioritized for conservation. Wang X. et al. (2021) revealed, through MaxEnt modelling, that precipitation in the warmest quarter (39.65%) and temperature stability were the core factors influencing the distribution of *A. spinulosa*, predicting that Luzhou (Sichuan) and Zunyi (Guizhou) are highly suitable regions; Lehmann et al. (2002) proposed a fern-diversity assessment method that provides a reference paradigm for integrative analyses.

Moreover, the omics technologies have further expanded research boundaries. Domzalska et al. (2017) revealed, through proteomics, that carbohydrate metabolism (enolase upregulated 2.8-fold) and fatty-acid metabolism (fatty-acyl-CoA reductase upregulated 4.2-fold) are the core regulatory pathways in the early stage of somatic-embryo (SE) initiation in *Cyathea delgadii*, identifying HSP70 and CDC48 as molecular markers for SE induction; Narayanan and Marimuthu (2016) discovered, through HPTLC, that gallic acid (Rf = 0.49) is a specific marker for *C. crinita* and quercetin (Rf = 0.75) is a common component of three *Cyathea* species. Chen J. H. et al. (2024) revealed, through meta-amplicon sequencing, that endophytic bacterial diversity is highest in the roots of *A. spinulosa* (Shannon index 4.2 ± 0.3). Flow cytometry can rapidly and accurately determine genome size and ploidy. Huang et al. (2020) determined that the genome size of *A. spinulosa* is c. 6,003.25 Mb; Wang et al. (2020) confirmed that *G. metteniana* is an allotetraploid group.

### 2.5 Comparison and prospects of technical methods

Different research techniques have their own advantages and disadvantages in resolution, data volume, cost, and applicable scenarios (Table 2). Traditional molecular markers are low-cost and suitable for preliminary screening of genetic diversity (Dong et al., 2019; Yang and Wang, 2025); genome-reduced sequencing is cost-effective and suitable for population-history reconstruction and cryptic-diversity mining (Yi et al., 2023; Wang et al., 2020; Xiao, 2019); whole-genome and transcriptome sequencing provide complete genetic information but are costly and suitable for in-depth genetic-mechanism dissection (Huang, 2022; Huang et al., 2025); integrative analysis of multi-omics and ecological models is the future core direction (Wang et al., 2020; Cheng et al., 2023). Current integration is hindered by (i) heterogeneous sequencing depths (30× vs. 100× genomes), (ii) absence of a pan-genome reference, and (iii) lack of standardized analytical pipelines (e.g., variant-calling parameters diverge among studies), emphasizing the need for consortia-based frameworks analogous to the Bird 10 K project.

**TABLE 2:** Comparison of major technical approaches used in genetic-diversity studies of the family Cyatheaceae.

Technology category	Representative technique	Resolution	Advantages	Limitations	Representative references
Morphological markers	Morphological trait observation and measurement	Low	Simple operation, directly linked to phenotypic adaptive features	Significantly affected by environmental factors, difficult to quantify genetic variation	Song, 2004; Cao et al. (2007), Yang et al. (2013)
Cytological markers	Chromosome karyotype observation and analysis	Low	Can reflect chromosomal-level genetic variation	Technically demanding, limited genetic resolution	Manton, 1950; Wang et al. (1997), Wei et al. (2025)
Biochemical markers	Isozyme/allozyme electrophoresis	Low–medium	Can reflect coding-region genetic variation, low cost	Limited number of polymorphic loci, low genetic resolution, susceptible to developmental-stage regulation	Yi et al. (2023), Wang and Guan (2011b)
Protein immunolabelling	Western blotting immunolabelling	Medium	Useful for distant-group comparison, reflects specific protein differences	Complex workflow, difficult antibody preparation	Conant and DeMaggio (1989), Domzalska et al. (2017)
Traditional molecular markers	RAPD, AFLP, ISSR, SSR, cpDNA sequence fragments	Medium	High polymorphism, mature technical systems, moderate cost for some methods	Limited genome coverage, some are dominant markers, variable repeatability	Wang et al. (2004), Li et al. (2010), Yang and Wang (2025), Zhou et al. (2009), Yang et al. (2025), Wang and Guan (2011a), Zhivotovsky (1999)
Chemical markers	FT-IR chemical-fingerprint analysis	Medium	Rapid, efficient, low-cost, non-destructive	Susceptible to environmental factors, indirect genetic-diversity indicator	Janakiraman and Johnson (2015)
Genome-reduced sequencing	RAD-seq, GBS	Medium–high	Can yield thousands to tens of thousands of SNPs, strong genome-wide representativeness	Reference-genome dependent, complex data-analysis pipeline	Yi et al. (2023), Zhou et al. (2009)
Whole-genome sequencing	PacBio/Nanopore + Hi-C	High	Provides complete information on genome structure, genetic variation and evolutionary history	High sequencing and analysis costs, large computational-resource demand	Huang et al. (2022), Huang et al. (2025)
Transcriptome sequencing	Illumina, PacBio Iso-Seq	High	Can reveal gene-expression profiles, functional genes and non-coding RNA features	Reflects only gene-expression status under specific conditions or in specific tissues	Huang et al. (2022), Shi et al. (2024)
Proteomics/metabolomics	Mass spectrometry, HPTLC	Medium–high	Can directly reflect functional-protein and metabolite diversity	Technically demanding, results susceptible to sample-preparation methods	Narayanan and Marimuthu (2016), Goh et al. (2007)
Microbiomics	16S rRNA/ITS meta-amplicon sequencing	Medium–high (community)	Can reveal symbiotic microbial diversity and host–microbe interactions	Limited species-level identification accuracy, functional inference requires experimental validation	Chen J. H. et al. (2024)

In the future, technologies such as single-cell sequencing, transcriptomics, and epigenomics are expected to be more widely applied, achieving breakthroughs in cell-specific expression regulation and environmental epigenetic regulatory mechanisms, and pushing research towards higher precision, deeper levels, and broader dimensions. Building on these methodological advances, we now examine how molecular data have resolved long-standing taxonomic controversies within Cyatheaceae.

## 3 Molecular phylogeny and taxonomic revision of Cyatheaceae

### 3.1 Delimitation of core monophyletic groups within the family and reconstruction of the systematic framework

The division of taxonomic units within Cyatheaceae has been debated for more than a century. Scholars disagree markedly over whether the family should be classified as one, three or six genera. The PPG I classification system proposed by Smith et al. (2006) recognizes only three genera and submerges the genus *Gymnosphaera* within *Alsophila*. Other researchers, using a variety of phylogenetic-analytical methods, have clarified a high-support, core, monophyletic division pattern for the entire family, dividing it into four stable clades: *Sphaeropteris*, *Alsophila* s.l., *Alsophila* sensu stricto, and *Gymnosphaera*, with *Sphaeropteris* as the basal most sister group of the other three clades and equilateral scales as the ancestral character. *Alsophila* S.S. and *Gymnosphaera* form a highly supported monophyletic branch (bootstrap >95%, posterior probability = 1.0) with marginal scales as the derived character (Korall et al., 2007; Li et al., 2010; Korall and Pryer, 2014).

Intensive sampling of Cyatheaceae in Southeast Asia, combined with morphological observation and spore-development-mechanism analyses, have confirmed that *Gymnosphaera* is markedly monophyletic and that it differs stably from *Alsophila sensu stricto*. in stipe color, indusial characteristics, and spore-development mode (64 vs. 16 spores per sporangium) (Korall et al., 2007; Dong and Zuo, 2018), advocating the restoration of *Gymnosphaera* as an independent genus and recognizing 43 species of *Gymnosphaera*, with East-Asian mainland and Madagascar identified as modern diversification centres. The genome study of Huang et al. (2022), the genomic data of Yi et al. (2023), the allopolyploid research of Wang et al. (2020), and the new hybrid species discovered by Schwartsburd et al. (2015) have further enriched the evolutionary-biology implications of the taxonomic framework.

### 3.2 Generic-level taxonomic revision: clarifying controversial species with molecular data

Molecular data provide key support for delimiting controversial species. Li et al. (2004), by analyzing chloroplast trnL intron and trnL-F intergenic spacer sequences of *Alsophila austroyunnanensis* and closely related species, found that its clusters with *Gymnosphaera* species in a highly supported monophyletic branch and recommended renaming it as *Gymnosphaera austroyunnanensis*, correcting the deviation of traditional morphological classification. Su et al. (2005) showed, through chloroplast atpB-rbcL intergenic spacer-sequence analysis of *Gymnosphaera* species in southern China, that they share multiple characteristic base variations with *Gymnosphaera* and support their inclusion in that genus.

Morigengaowa et al. (2019) using a combination of reduced-genome and morphological evidence, revealed significant species-diversity differentiation within *S. brunoniana*. Wang and Guan (2011b) and Cao et al. (2007) clarified the genetic structure and phylogeographical pattern of *S. brunoniana* through AFLP markers and cpDNA-sequence analysis. Zhong et al. (2024) confirmed that the chloroplast psbA-trnH sequence can effectively distinguish closely related controversial species. The chloroplast-genome analysis of Chen X. et al. (2024) and the novel nuclear SSR markers developed by Ruan et al. (2017) provide multi-level molecular evidence for delimiting controversial species.

### 3.3 Re-evaluation of the phylogenetic significance of morphological characters

Accumulation of molecular data has promoted the re-evaluation of the phylogenetic significance of morphological characters. Korall et al. (2007), through ancestral-state reconstruction, found that scale morphology (equilateral vs. marginal) has key phylogenetic significance in major-group delimitation; indusial morphology has a weak phylogenetic signal and can provide support only for the delimitation of some sub-clades. Li et al. (2007) analyzed 42 phenotypic traits of *G. metteniana* and showed that reproductive-organ-related traits, such as the number of scale-margin serrations and indusium diameter, vary little among populations and are significantly positively correlated with the degree of genetic differentiation shown by cpDNA sequences, whereas vegetative-organ traits such as frond length, have high phenotypic plasticity, are regulated by environmental factors, and show no significant correlation with genetic data, revealing that taxonomic studies should give priority to more conservative reproductive-organ traits and carry out comprehensive evaluation combined with molecular data.

Cao et al. (2007) found that spore-surface ornamentation and size are stable at the generic level and can be used as auxiliary taxonomic evidence. Narayanan and Marimuthu (2016) through HPTLC fingerprint analysis, found that chemical morphological characters such as phenolic compounds have certain taxonomic value at the generic level. The FT-IR spectroscopic analysis of Janakiraman and Johnson (2015) revealed that spectral characteristics of ethanol extracts from gametophytes and sporophytes can be used for group delimitation. The proteomics study of Domzalska et al. (2017) found that differentially expressed proteins in stem-segment explants are related to somatic embryogenesis, providing a new perspective for linking morphogenetic mechanisms with phylogeny. These phylogenomic patterns align with the Gondwanan break-up timeline, supporting a vicariance-driven diversification scenario rather than recent long-distance dispersal, and thus link micro-evolutionary processes to continental-scale biogeography.

## 4 Core findings on genetic diversity in Cyatheaceae

### 4.1 Plastid-genome diversity

The chloroplast genome serves as an important carrier for genetic-diversity studies. Its structural features and variation patterns provide key evidence for systematics and species identification. Chen J. H. et al. (2024) found that the chloroplast genome of *A. spinulosa* is 156,196 bp long and possesses the typical quadripartite structure. Ma et al. (2020) confirmed that gene order is consistent with that of most ferns and is highly conserved. Comparative-genomics studies reveal that, on the basis of overall conservation, species-specific structural variants exist in the chloroplast genomes of Cyatheaceae. Hu et al. (2023) found that *S. brunoniana* lacks the trnV-UAC gene and that the small single-copy (SSC) region is markedly longer than in other species. Zhu et al. (2021) found that the number, type, and distribution of SSRs in cpDNA differ significantly among genera and can serve as effective molecular markers for generic identification. Wang Z. J. et al. (2021) further supplemented evidence for inter-generic genome-structure variation through analysis of the chloroplast genome of *Alsophila latebrosa*.

Screening of genes with high nucleotide diversity provides support for DNA-barcode development. Hu et al. (2023) analyzed chloroplast genomes of eight Cyatheaceae species and found that nucleotide diversity (π) of eight genes, including *atpI* and *ycf2,* was >0.015, significantly higher than the genome-wide average, and proposed that the combination of the trnG-trnR spacer and *atpB* gene can be used effectively for population-level identification of *S. brunoniana*. Zhong et al. (2024) verified the effectiveness of the psbA-trnH spacer in distinguishing closely related species. Zhou et al. (2009) pointed out that the combined use of cpDNA fragments and nuclear markers (SSR, RAD-seq SNP) can markedly improve DNA-barcode identification efficiency and accuracy.

### 4.2 Cryptic diversity and hybridisation in the nuclear genome

Nuclear-genome information provides deep support for revealing species differentiation and evolutionary processes. The widespread existence of cryptic diversity is an important reason why species diversity in Cyatheaceae has been underestimated. Yi et al. (2023) conducted a genome-level study using RAD-seq technology, performing phylogenetic and population-genomic analyses on 16 scaly Cyatheaceae species from China and Vietnam. The results showed that each of the three widespread *Gymnosphaera* species possessed cryptic diversity, each species being composed of two highly structured lineages that may correspond to cryptic taxa. Morigengaowa et al. (2019) combined reduced-genome and morphological evidence and revealed significant species-diversity differentiation within *S. brunoniana*, correcting the previous perception that it was a single species.

Hybridization and introgression play complex roles in the evolution of Cyatheaceae. Yi et al. (2023) detected widespread hybridization and introgression signals among both closely related species and between *Alsophila* and *Gymnosphaera* genera using ABBA-BABA tests, with introgressed genes being enriched in adaptive pathways such as “stress response” and “xylem development”. Wang et al. (2020) confirmed that allopolyploid speciation, accompanied by gene flow, is an important pathway for new-species formation; Schwartsburd et al. (2015) discovered a new hybrid species in Brazilian tree-ferns, corroborating the universality of hybridization. However, when parental genetic divergence is too large, hybrid progeny may suffer from chromosomal-segregation disorders and reduced fertility, indicating that hybridization has both “creative” and “destructive” attributes.

### 4.3 Population genetic structure and historical dynamics

Geographical isolation is a key factor driving population genetic differentiation in Cyatheaceae. Hu et al. (2023) showed that genetic distance is significantly positively correlated with geographical distance in *S. brunoniana*, fitting the “isolation-by-distance” model. The Qiongzhou Strait causes high genetic differentiation between Hainan and mainland *A. spinulosa* populations, whereas no significant differentiation was detected among *G. podophylla* populations spanning the strait, inferred to be related to continuous gene flow during geological history (Su et al., 2005; Su et al., 2004b); complex topography in south-west China also drives population differentiation in Cyatheaceae (Zhu and Peng, 2006).

Quaternary glacial–interglacial cycles have exerted profound effects on the distribution patterns and genetic diversity of Cyatheaceae. Areas such as Yunnan, Mount Emei in Sichuan, Diaoluo Mountain in Hainan, and coastal Fujian in China may have served as glacial refugia (Loiseau et al., 2020; Xie et al., 2022; Wang X. et al., 2021; Wang Z. et al., 2021). These refugial populations still retain high genetic diversity and many private alleles or haplotypes. Huang (2022) and Huang et al. (2022) revealed, through PSMC modelling, that *A. spinulosa* experienced two population-size contractions during the Last Glacial Maximum and the early Holocene, which is a key reason for the currently low genetic diversity of many wild populations. Wang Z. et al. (2021) further verified the habitat suitability of these refugial regions.

At local scales, population genetic structure is significantly affected by life-history traits. Ramírez-Barahona and Eguiarte (2015) found that *Alsophila firma* exhibits strong spatial genetic structure, which is directly related to life-history traits such as limited gametophyte dispersal and widespread clonal growth in ferns; Suárez-Santiago et al. (2024) confirmed that clonal reproduction intensifies local spatial genetic structure and reduces population genetic diversity in *Culcita macrocarpa*.

### 4.4 Adaptive genetic diversity

Dissecting adaptive genetic diversity is central to revealing mechanisms of environmental adaptation in species. The arborescent morphology of Cyatheaceae is closely related to unique functional-gene families. Huang et al. (2025) conducted a genome-wide identification of the *bHLH* transcription-factor family in *A. spinulosa* and found 186 *bHLH* genes, a number significantly higher than in herbaceous fern species. Transcriptome analysis and co-expression-network construction showed that these genes are specifically highly expressed in xylem and are highly co-expressed with key enzyme genes in the lignin-biosynthesis pathway, implying that they play a core regulatory role in stem development and lignification (Huang et al., 2020). Huang (2022) further revealed the evolutionary history of xylem-development-related genes through genome deciphering.

Diversity of stress-resistance and light-adaptation genes provides a guarantee for Cyatheaceae to cope with complex environments. Hong et al. (2022) analyzed the function of the ASR gene family in the *A. spinulosa* transcriptome and found that the frequency of the ASR2 allele in drought-habitat populations was significantly higher than in humid-habitat populations, and that the expression level of this allele increased most significantly under drought stress. Antioxidant enzyme genes such as catalase (CAT) and peroxidase (POD) are key functional genes responding to high-temperature stress, and five classes of photoreceptor genes and key members of their signaling pathways were also identified. Peng et al. (2024) supplemented the stress-resistance gene resources of *S. brunoniana* and *A. latebrosa* through multi-organ full-length transcriptome analysis. The genetic diversity aspects related to stressors in Cyatheaceae are given in Table 3.

**TABLE 3:** Genetic diversity aspects related to biotic and abiotic stress in Cyatheaceae.

Aspect	Biotic stress (e.g., pathogens, herbivores, competition)	Abiotic stress (e.g., climate change, drought, temperature)
Genetic Response	Pathogen recognition genes; secondary metabolite biosynthesis (e.g., alsophilin) ( Narayanan and Marimuthu, 2016; Huang et al., 2025 )	Stress-responsive transcription factors (e.g., *bHLH*); antioxidant enzymes (CAT, POD) ( Huang et al., 2025; Hong et al., 2022 )
Adaptive Mechanism	Chemical defense; microbiome-mediated resistance ( Chen X. et al., 2024; Liu, 2012 )	Phenotypic plasticity; gene expression modulation; epigenetic regulation ( Hong et al., 2022 ; Peng et al., 2024 )
Diversity Pattern	Higher diversity in pathogen-rich, humid tropics ( Krishnan and Rekha, 2021; Korall and Pryer, 2014 ); local adaptation to pests	Higher diversity in stable refugia ( Wang X. et al., 2021; Wang Z. et al., 2021 ); reduced diversity in fragmented habitats ( Wei et al., 2021; Wang T. et al., 2021 )
Conservation Implication	Preserve populations with unique chemical and resistance traits ( Narayanan and Marimuthu, 2016; Goh et al., 2007 )	Prioritize refugial populations with climate-resilient alleles ( Wang Z. J. et al., 2021; Wang and Guan, 2011a )
Example Study Focus	Endophytic community influence: antifungal compound variation ( Chen J. H. et al., 2024; Huang et al., 2025 )	Drought/heat-responsive gene expression; chloroplast genome variation ( Hong et al., 2022; Hu et al., 2023 )

Variation in secondary-metabolism-related genes endows Cyatheaceae with important ecological and medicinal value. Huang et al. (2025) and Huang (2022), through genome annotation and metabolome-association analysis, clarified the complete biosynthesis pathway of the specialized phenolic compound alsophilin in *A. spinulosa* and identified key enzyme genes such as PAL, C4H, and 4CL. These genes show obvious allelic variation and expression-level differences. Alsophilin has antifungal activity, and its accumulation is significantly negatively correlated with population disease and pest incidence (Huang, 2022; Huang et al., 2022; Huang et al., 2025). The complex rheological properties of the water-soluble extract from fronds of *G. podophylla* and chromatographic analysis and target docking of steroidal compounds in Cyatheaceae provide new directions for medicinal-value development (Goh et al., 2007; Janakiraman et al., 2023). The fingerprint study of Liu (2012) provides technical support for standardized identification of secondary metabolites.

### 4.5 Reproductive system, microbiome, and genetic diversity

The reproductive system profoundly affects genetic diversity in Cyatheaceae by regulating the generation and transmission of genetic variation. Suárez-Santiago et al. (2024) found that clonal reproduction is widespread in *Culcita macrocarpa* and is especially prominent in island populations, leading to significantly reduced within-population genetic diversity. Pelosi and Sessa (2021) pointed out that mating system and growth habit have significant effects on fern population genetic diversity (% P) and population structure (FIS, FST). Pelosi and Sessa (2021) confirmed that high selfing rates significantly reduce population genetic diversity and cause inbreeding depression. Wang et al. (2012) found that *A. spinulosa* can adjust reproductive allocation depending on environmental suitability: in suitable habitats, resources are preferentially allocated to sexual reproduction to increase offspring genetic variation, whereas instressful environments, asexual reproduction dominates to maintain population size rapidly.

The microbiome is closely related to host genetic diversity. Chen X. et al. (2024) used high-throughput sequencing to demonstrate significant tissue specificity in the endophytic microbial communities of *A. spinulosa* roots, stems, and leaves. They found that community composition and network complexity correlate with host tissue function. The authors infer that the host’s genetic background indirectly regulates its phenotypic expression and environmental adaptability by shaping tissue-specific microenvironments that determine microbial colonization. Although correlative studies associate root-associated Burkholderia with drought tolerance (Chen J. H. et al., 2024), experimental inoculations and gnotobiotic systems are needed to establish causality. Such validation could open microbiome-assisted reintroduction protocols analogous to those developed for orchids (Smith and Read, 2010). Specifically, functional validation approaches such as controlled microbial inoculation experiments, gnotobiotic (sterile) growth systems, and microbiome transplantation have been successfully used in angiosperms to demonstrate direct effects of microbial consortia on drought tolerance, nutrient acquisition, and pathogen resistance (Trivedi et al., 2020). These approaches allow host phenotypic responses to be directly attributed to defined microbial taxa or functional groups, thereby moving beyond correlation.

Recent evidence suggests that root-associated bacterial taxa, particularly members of *Burkholderia*, *Pseudomonas*, and *Actinobacteria*, can enhance abiotic stress tolerance by modulating phytohormone signaling, osmolyte accumulation, and antioxidant activity (Vandenkoornhuyse et al., 2015). In ferns and other early-diverging land plants, symbiotic microorganisms have also been shown to influence nutrient uptake and stress resilience, indicating that microbiome-mediated adaptation is likely an evolutionarily conserved strategy (Delaux et al., 2015).

The content and composition of medicinally active secondary metabolites in Cyatheaceae vary significantly among species and among populations within species, and the degree of variation is positively correlated with geographical isolation distance and habitat heterogeneity (Wang, 1993; Dong and Zuo, 2018; Liu, 2012), reflecting the diversity of the genetic basis and of gene-expression-regulation networks. Understanding reproductive and microbial influences on genetic variation sets the stage for dissecting how historical and contemporary geography shape diversity patterns (Section 5).

## 5 Geographic patterns and driving mechanisms of genetic diversity in Cyatheaceae

### 5.1 Geographic-pattern characteristics

On a global scale, hotspots of species diversity and genetic diversity are concentrated in the American tropics (Amazon basin, Central-American cloud forests) and in the tropical and subtropical Asia–Pacific regions (Southeast-Asian archipelagos, southern China, north-eastern Australia) (Mo and Liu, 2004; Korall and Pryer, 2014), where the climate is warm and humid, habitats are diverse and large-scale glacial destruction has not occurred (Korall and Pryer, 2014; Suárez-Santiago et al. (2024)). The African continent shows markedly depauperate diversity because of climatic aridification and habitat fragmentation (He et al., 2022). We acknowledge that the present synthesis is inevitably skewed toward China and Southeast Asia (≈70% of cited studies), whereas African and Neotropical Cyatheaceae remain under-represented (≈8%). This geographic gap hinders global meta-analyses of diversification drivers and may mask continent-specific conservation priorities. The Malay Archipelago is the global center of speciation and genetic diversity of Cyatheaceae (Korall and Pryer, 2014). Sri Lanka has formed unique genetic lineages due to long-term geographical isolation (Ranil et al., 2017), and the Western Ghats of India likewise exhibit high genetic specificity in Cyatheaceae (Narayanan and Marimuthu, 2016; Krishnan and Rekha, 2021).

At the regional scale, southern China and Southeast Asia can be divided into four typical areas:

1. South China (Hainan Island, Xishuangbanna in Yunnan) has high habitat connectivity and a humid, stable climate; Cyatheaceae populations generally show high genetic diversity and low among-population genetic differentiation (Wang and Guan, 2011a; Su et al., 2005; Wang Z. J. et al., 2021).
2. South-west China (Chishui River basin, Hengduan Mountains) has strong habitat heterogeneity. Genetic diversity shows a “core–edge” gradient distribution (Wang T. et al., 2021; Wang and Guan, 2011a): core areas (middle-lower reaches of the Chishui River basin, *etc.*) have the highest genetic diversity, whereas edge areas (high-elevation slopes, *etc.*) gradually decrease with habitat degradation (Wang X. et al., 2021; Yang et al., 2013).
3. Lingnan region (Guangdong, southern Guangxi) has severe habitat fragmentation, populations show “insular” distribution, genetic-diversity levels are low, and among-population differentiation is significant (Wei et al., 2021; Hu et al., 2023).
4. Distribution areas of rare and endemic species (rock crevices, high-humidity gullies) have specialized habitats, narrow distribution ranges, extremely low within-population genetic diversity, are extremely sensitive to external interference, and have very weak population-recovery ability (Zhou et al., 2008; Li et al., 2004).

### 5.2 Driving mechanisms

The pattern of genetic diversity is shaped primarily by temperature, precipitation, and habitat connectivity. Specifically, extreme low temperatures and high thermal fluctuations inhibit spore germination and seedling survival, while the stable, warm conditions (20 °C–25 °C) characteristic of tropical and subtropical regions provide the ideal basis for the formation of genetic diversity hotspots (Wei et al., 2021; Wang Z. et al., 2021; Korall and Pryer, 2014). Precipitation in the warmest quarter regulates Cyatheaceae growth and spore-dispersal efficiency by affecting water availability; insufficient precipitation leads to habitat aridification and reduces population genetic diversity (Wang X. et al., 2021; Wang Z. et al., 2021). Urbanization expansion and agricultural reclamation cause habitat “patchiness”, hindering spore dispersal and forming “genetic islands”; in the fragmented habitat of the Lingnan region, the gene-flow rate of Cyatheaceae populations is only one-third that in the continuous habitat of South China (Wei et al., 2021; Wang Z. J. et al., 2021).

Genetic variation accumulation is modulated directly by the mating system and dispersal ability. This is evident in outcrossing species, which are characterized by elevated intra-population genetic diversity and diminished inter-population genetic differentiation (Wang et al., 2004; Wang et al., 2012), whereas selfing or inbreeding populations easily limit genetic-variation richness (Pelosi and Sessa, 2021). Spore-dispersal efficiency is constrained by wind speed, topography, and connectivity of suitable patches; complex topography in south-west China hinders spore dispersal across regions, whereas flat terrain and stable monsoon climate in South China promote among-population gene homogenization (Su et al., 2005; Wang and Guan, 2011a). Quaternary glacial–interglacial cycles have exerted decisive effects on the genetic pattern: suitable habitats contracted during glacial periods, forcing Cyatheaceae to retreat into “refugia”; leading-edge populations after glacial expansion suffered from founder effects and genetic drift, resulting in significantly lower genetic diversity than in core refugial populations (Su et al., 2004a; Wang and Guan, 2011a; Korall and Pryer, 2014; Suárez-Santiago et al., 2024).

Biotic factors also participate in regulating the genetic pattern. In inter-specific competition, expansion of bamboo (*Phyllostachys edulis*) suppresses Cyatheaceae growth by resource competition, leading to reduced effective population size and accelerated loss of genetic diversity (Qu et al., 2020). Hybridization between closely related species has dual effects: it can provide raw material for species evolution (Wang et al., 2020) but may also cause contamination of pure gene pools and threaten species’ genetic integrity (Yi et al., 2023). With geographic and ecological drivers clarified, we have explored how genetic insights can be translated into practical conservation strategies (Section 6). Endophytic bacterial and fungal communities indirectly regulate population growth and reproductive efficiency by affecting host nutrient uptake and stress tolerance (Chen X. et al., 2024).

Genetic characteristics themselves constrain diversity accumulation. Cyatheaceae generally possess extremely large genomes (C-value 10–30 pg), with low genome-replication and repair efficiency and slow gene-mutation accumulation rates (Narayanan and Marimuthu, 2016; Huang et al., 2020); the gene-mutation rate of *A. spinulosa* is only one-fifth that of angiosperms (Narayanan and Marimuthu, 2016; Janakiraman et al., 2023). Although polyploidization can increase genetic diversity, it may cause reproductive barriers, and phenomena such as repeat-sequence expansion and gene silencing significantly reduce the effective accumulation of genetic variation (Wang et al., 2020; Wang et al., 2004; Wei et al., 2025). The special gene-expression regulation of transcription-factor families such as *bHLH* may also indirectly affect population adaptive genetic variation (Huang et al., 2025).

## 6 Applications of genetic-diversity research in the conservation of Cyatheaceae

### 6.1 Guiding *ex-situ* conservation and precise germplasm preservation

Genetic-diversity research provides core scientific evidence for *ex-situ* conservation. For example, RAD-seq revealed two cryptic lineages within *G. podophylla* (Yi et al., 2023); both are now maintained in separate living collections at the Xishuangbanna Tropical Botanical Garden, illustrating direct translation of genomic data into conservation units. Germplasm collection should prioritize refugial populations that have retained more adaptive genes and unique genetic lineages (Wang X. et al., 2021; Wang and Guan, 2011a). Sampling must meet the minimum effective population size (≥50 reproductive individuals) and cover the main genetic lineages within populations through molecular markers such as SSR and AFLP (Pelosi and Sessa, 2021; Frankham et al., 2002; Wang and Guan, 2011b). For groups with cryptic species differentiation, each cryptic lineage should be treated as an independent conservation unit (Morigengaowa et al., 2019; Wang and Guan, 2011a). Existing *ex-situ* conservation needs to supplement germplasm from marginal populations with low genetic diversity but high evolutionary uniqueness, adopting a “natural habitat + *ex-situ*” dual-repository conservation model (Zhan, 2022b); spatial-distribution data of genetic diversity can be used to identify conservation-gap areas precisely (Wei et al., 2021; Zhan, 2022a).

### 6.2 Assisting accurate species delimitation and scientific designation of conservation units

Molecular-marker techniques provide effective tools for accurate species delimitation and conservation-unit designation. Chloroplast-genome fragments (psbA-trnH, trnL-F), nuclear SNPs, and SSR markers can accurately distinguish closely related species, cryptic species, and natural hybrid individuals (Dong et al., 2019; Zhong et al., 2024; Hu et al., 2023), providing support for defining conservation targets. By combining degree of genetic differentiation (FST values), differences in adaptive genes and gene-flow intensity, populations can be divided into evolutionarily significant units (ESUs) and management units (MUs) (Pelosi and Sessa, 2021; Wang and Guan, 2011a); ESUs with FST >0.25 require independent conservation strategies, whereas MUs with Nm < 1 should be prioritized for habitat-connectivity restoration (Yi et al., 2023; Wang and Guan, 2011a).

### 6.3 Dissecting endangerment mechanisms and scientifically assessing extinction risk

Molecular markers provide a powerful tool for elucidating the mechanisms behind species endangerment and for assessing extinction risk. By quantifying key parameters such as allelic richness and heterozygosity, they directly measure a population’s adaptive evolutionary potential, with lower genetic diversity strongly correlating with a greater risk of extinction (Zhou et al., 2008; Morigengaowa et al., 2019). Methods such as allelic-frequency distribution and heterozygosity-deviation tests can detect whether populations have experienced genetic bottlenecks or long-term inbreeding (Morigengaowa et al., 2019; Wang et al., 2012). Interspecific hybridization and introgression pose threats to some endangered species (Yi et al., 2023; Wang et al., 2020), and molecular markers are needed to identify hybrid individuals and to develop isolation-protection measures (Pelosi and Sessa, 2021). Combined with population-ecology research (Song, 2004), “genetic-factor–ecological-factor” synergistic endangerment-mechanism dissection can be realized. For example, the superposition of population-size reduction caused by bamboo expansion and genetic drift will accelerate the loss of genetic diversity in Cyatheaceae (Qu et al., 2020). Integrating genetic-diversity parameters with habitat-suitability models can build a “genetic–ecological” two-dimensional risk-assessment system (Wang T. et al., 2021).

### 6.4 Optimizing nature-reserve networks and monitoring conservation effectiveness

Genetic-diversity data are essential for optimizing reserve networks and monitoring conservation outcomes. A key strategy involves overlaying genetic-diversity hotspots with areas predicted as highly suitable by species distribution models. Identifying these “double-high” regions—where high genetic diversity coincides with high habitat suitability—provides a scientific basis for expanding existing reserves or selecting new reserve sites (Wang Z. et al., 2021; Yang et al., 2013; Wang Z. J. et al., 2021). For marginal populations not included in protected areas, conservation plots or ecological corridors need to be established (Wei et al., 2021; Zhan, 2022b). A long-term monitoring system based on genetic parameters should be built to detect indicators such as allelic richness and gene-flow intensity regularly so as to evaluate the effectiveness of conservation measures (Xie et al., 2022; Yang et al., 2013; Wang and Guan, 2011a). Combined with transcriptomics, expression changes in stress-response and adaptive genes can be monitored to provide early warning of environmental-change impacts (Hong et al., 2022; Peng et al., 2024). Lineage-tracking techniques based on cpDNA sequences can evaluate the genetic integrity of *ex-situ* conserved populations and gene-exchange situations after field planting (Zhan, 2022a; Ma et al., 2020).

### 6.5 Integrating phenotype–genotype associations for habitat-matching conservation

Integrative analysis linking phenotypic variation and genetic differentiation can realize precise habitat regulation. For *in-situ* conservation, threshold values of key habitat factors must be clarified, e.g., canopy transmittance 30%–50%, soil pH 5.5–6.5 (Xie et al., 2022; Chen J. H. et al., 2024), and water conditions should be regulated in marginal areas (Wang X. et al., 2021). For *ex-situ* conservation site selection, a “habitat-similarity index” should be established, giving priority to areas with an index ≥80% (Wei et al., 2021; Wang T. et al., 2021), and survival rates can be improved by combining light-adaptation and stress-resistance related gene-expression characteristics (Hong et al., 2022; Peng et al., 2024). Combining conserved phenotypic traits with genetic-diversity parameters of SSR markers can provide rapid feedback on how habitat change affects population genetic adaptability (Xie et al., 2022; Cao et al., 2007; Yang et al., 2013).

### 6.6 Supporting sustainable utilisation and optimization of artificial-propagation techniques

Under the premise of strict wild-population protection, genetic-diversity research can support sustainable resource utilization. Populations with high contents of functional compounds can be screened based on genetic background for moderate development of medicinal and healthcare resources (Narayanan and Marimuthu, 2016; Goh et al., 2007; Liu, 2012). A “artificial cultivation–component extraction–profit feedback to conservation” model can be established to improve the economic feasibility of conservation (Wang Z. J. et al., 2021). In artificial propagation, genetic-marker-assisted parental selection should give priority to crossing individuals from different geographical populations or lineages to ensure sufficient variation in progeny (He et al., 2022; Yi et al., 2023); conditions such as spore disinfection and medium formulation should be optimized, and native temperature, humidity and photoperiod should be simulated to improve the genetic adaptability of artificially propagated individuals and their survival rate after field planting (Mo and Liu, 2004; Domzalska et al., 2017). For polyploid species, cytological identification is needed to avoid reproductive barriers caused by abnormal chromosome segregation (Wang et al., 1997; Wang et al., 2020).

## 7 Research prospects

“Cyatheaceae, an ancient tree-fern lineage surviving from the Mesozoic, offers both important theoretical value and urgent practical significance for omics research and genetic-diversity conservation. Although series advances have been achieved in genomics, transcriptomics and related fields, four core challenges remain: (i) taxonomic and geographical coverage imbalance leading to a lack of global genetic-pattern cognition; (ii) insufficient depth of technology application and weak multi-omics integration, making it difficult to dissect molecular-network-level genetic regulatory mechanisms; (iii) broken genetic–function–phenotype linkages, so that core driving factors of adaptive evolution are still unclear; (iv) absence of long-term dynamic monitoring, resulting in a lag in translating research results into conservation practice. We recognize several dataset limitations: (i) taxon sampling is heavily skewed to 14 Chinese species, leaving >600 Cyatheaceae species genetically unexplored; (ii) most population studies sample <30 individuals, limiting robust allele-frequency estimates; (iii) only one chromosome-level reference genome is available, impeding pan-genome assembly; and (iv) African and Neotropical lineages are represented by <10% of omics studies, creating geographic blind spots that may conceal unique evolutionary trajectories. To promote high-quality development in this field, future efforts should focus on the following four directions:

1. Short-term (≤3 years): Expand omics-research coverage. Relying on low-cost genome-reduced-sequencing technologies such as RAD-seq and GBS, fill the genetic blanks for the majority of species in the genus *Sphaeropteris* and for minor lineages within the family, and raise research coverage from <5% to >30% of core taxa; strengthen international co-operation, carry out cross-continental comparative studies, focus on dissecting population genetic structure in global biodiversity hotspots such as the Amazon basin and African arid regions, reveal the shaping effects of macro-processes such as Gondwanan break-up and inter-continental climatic differentiation on genetic patterns, and construct a global-scale genetic-diversity pattern.
2. Mid-term (3–5 years): Strengthen multi-omics integration and functional validation. Expand the species range of whole-genome and transcriptome sequencing, build an integrative analytical framework of “genome–transcriptome–epigenome–metabolome–microbiome”; mine key genes and pathways regulating genetic diversity with bioinformatic tools such as WGCNA and GWAS, verify biological functions of stress-resistance and secondary-metabolism-synthesis genes by CRISPR–Cas9 gene editing, clarify molecular pathways through which genetic variation affects phenotypic adaptability, and link the causal chain of “genotype–phenotype–environment”.
3. Long-term (>5 years): Deepen the integration of conservation genetics with practice. Carry out deeper conservation-genetics studies on endangered species, combining genomics, population genetics and ecological analyses to assess patterns of genetic diversity, population structure, gene flow and deleterious-mutation distribution, and to reveal endangerment mechanisms and evolutionary potential; incorporate key information such as genetic-diversity hotspots, cryptic lineages and ESUs into nature-reserve-network optimization planning, formulate precise sampling strategies for *ex-situ* conservation, and establish standardized procedures for “natural-habitat + *ex-situ*” dual-repository conservation; develop rapid field-population monitoring technologies based on molecular barcoding and build a “genetic–ecological” two-dimensional endangerment-risk-assessment system.
4. Continuous: Innovate technical methods and research dimensions. Introduce single-cell sequencing to dissect gene-expression dynamics during spore development, use transcriptomics to locate tissue-specific expression patterns of stress-resistance and metabolism-related genes; carry out epigenetic studies to reveal the regulatory roles of DNA-methylation and other epigenetic marks under environmental stress; strengthen comparative omics studies between Cyatheaceae and closely related fern families, uncover their unique evolutionary characteristics, and provide new perspectives for fern classification and evolutionary research.

## 8 Conclusion

In conclusion, this review synthesizes the significant progress in multi-omics research on Cyatheaceae, highlighting its profound genetic diversity and evolutionary adaptations. A critical insight from recent studies is the elucidation of genetic mechanisms underpinning the family’s resilience to various biotic and abiotic stresses, including climate fluctuations, habitat fragmentation, and competitive pressures. Key findings involve stress-responsive gene families, adaptive hybridization, and the role of endophytic microbiomes. However, challenges such as taxonomic bias and fragmented data integration persist. Future research must prioritize multi-omics integration and functional validation to decode stress-adaptation networks fully. Ultimately, leveraging this genetic knowledge is imperative for developing robust, science-based conservation strategies to ensure the survival and evolutionary potential of these ancient ferns in a rapidly changing and increasingly stressful world. Linking rapidly eroding genetic diversity (Section 4.3) with measurable adaptive loci (Section 4.4) and actionable conservation units (Section 6.2) underscores an urgent need to couple genomic surveillance with immediate habitat protection, ensuring that evolutionary potential is not lost before it is described.
